# Assessing quality of maternity care in Hungary: expert validation and testing of the mother-centered prenatal care (MCPC) survey instrument

**DOI:** 10.1186/s12978-017-0413-3

**Published:** 2017-11-16

**Authors:** Nicholas Rubashkin, Imre Szebik, Petra Baji, Zsuzsa Szántó, Éva Susánszky, Saraswathi Vedam

**Affiliations:** 1 Departments of Global Health Sciences and Obstetrics, Gynecology, and Reproductive Sciences, University of California at San Francisco, Mission Hall, Box 1224, 550 16th Street, Third Floor, San Francisco, California 94158 USA; 2 Institute of Behavioral Sciences, Semmelweis University, VIII. Nagyvárad tér 4. XX. Em, Budapest, H-1089 Hungary; 3 Department of Health Economics, Corvinus University of Budapest, Fővám tér 8. Main Building Room E113, Budapest, 1093 Hungary; 4 The Birth Place Lab, Faculty of Medicine, The University of British Columbia, Vancouver, Canada; 5 Midwifery Program | Department of Family Practice, Suite 320 - 5950 University Boulevard, Vancouver, BC V6T 1Z3 Canada

**Keywords:** Questionnaire, Validation, Respectful maternity care, Informal payments, Hungary

## Abstract

**Background:**

Instruments to assess quality of maternity care in Central and Eastern European (CEE) region are scarce, despite reports of poor doctor-patient communication, non-evidence-based care, and informal cash payments. We validated and tested an online questionnaire to study maternity care experiences among Hungarian women.

**Methods:**

Following literature review, we collated validated items and scales from two previous English-language surveys and adapted them to the Hungarian context. An expert panel assessed items for clarity and relevance on a 4-point ordinal scale. We calculated item-level Content Validation Index (CVI) scores. We designed 9 new items concerning informal cash payments, as well as 7 new “model of care” categories based on mode of payment. The final questionnaire (*N* = 111 items) was tested in two samples of Hungarian women, representative (*N* = 600) and convenience (*N* = 657). We conducted bivariate analysis and thematic analysis of open-ended responses.

**Results:**

Experts rated pre-existing English-language items as clear and relevant to Hungarian women’s maternity care experiences with an average CVI for included questions of 0.97. Significant differences emerged across the model of care categories in terms of informal payments, informed consent practices, and women’s perceptions of autonomy. Thematic analysis (*N* = 1015) of women’s responses identified 13 priority areas of the maternity care experience, 9 of which were addressed by the questionnaire.

**Conclusions:**

We developed and validated a comprehensive questionnaire that can be used to evaluate respectful maternity care, evidence-based practice, and informal cash payments in CEE region and beyond.

## Plain English summary

Women in Hungary and in the CEE region report negative experiences with pregnancy care. It is unknown how common these experiences are. High numbers of women pay their obstetricians with informal cash payments, sometimes called “tips”, in order to obtain higher quality care. We don’t know if, when women pay informally, they actually get higher quality care.

In order to quantitatively explore the experience of quality maternity care in Hungary, we assembled a multi-disciplinary expert panel to adapt English-language maternity care surveys to the Hungarian context. We instructed the experts to think broadly about all aspects of care that may be important to women.

Generally, the experts found that English-language surveys could be easily adapted, and they helped us narrow the number of survey questions from 155 to 117. Because the informal payment, or “tip” system, is specific to Hungary, the experts developed new questions using Hungarian words to represent this practice. We then tested all questions on two groups of post-partum Hungarian women who use the internet: a random, representative sample and another group recruited from online maternity care forums.

We found that the new questions about informal cash payments made sense to women and that women reported more positive experiences with care when they paid informally. Women’s responses to an open-ended question revealed that we addressed the majority of care dimensions that mattered to them.

In conclusion, we developed a survey to comprehensively explore the maternity care experience in Hungary. Our survey process and questions may be useful to explore the maternity systems of surrounding countries.

### Article summary

#### Strengths

We used a rigorous process to develop and validate a patient survey instrument that can evaluate women’s experiences in the Hungarian maternity care system.

No other survey has explored the connection between informal cash payments and quality of maternity care in the CEE region.

#### Limitations

Survey development could have employed more active users of the maternity system. Some care dimensions important to women were not addressed by the survey.

## Background

Person-centered care has been associated with engendering the most optimal relationship between patient and provider in all medical specialties [1]. Balanced sharing of information, individualized care plans, and continuous emotional support are elements that have been shown to improve birth care outcomes and increase satisfaction with the birth experience [2, 3]. The World Health Organization’s (WHO) vision for quality of care for pregnant women and newborns mandates both the provision of evidence-based medical services and curation of the maternal experience [4, 5]. However, in a systematic review of 65 studies across 34 countries Bohren and colleagues confirmed that few tools exist to measure the experience of respect or mistreatment in maternity care [6].

While the CEE region demonstrates generally favorable maternal health indicators [7], Miteniece et al.’s systematic review of 20 investigations into the region’s quality of maternity care confirmed the need to also assess the professional, technical, and informational aspects of maternity care [8]. Birth care providers are often trained with outdated curricula [9], resulting in overapplication of non-evidence-based techniques. Doctor-patient communication is often poor, with providers lacking the skills to interpret what mothers need during pregnancy care [10, 11]. Women themselves play an important role in the doctor-patient interaction during pregnancy, including uptake of information, optimizing health behaviors, and adherence to care. However, there is scarce information about whether women in the CEE region have autonomy over their childbearing experiences. Mitenice et al. conclude that the evidence on these and other aspects of quality maternity care in the CEE region are derived in large part from qualitative designs, and few studies provide nationally representative data [8].

To date, there have been conflicting quantitative investigations of the quality of maternity care in Hungary. In a 1998 survey of academic obstetric departments, Hagymasy found high levels of “family-centered obstetrics”, defined as involvement of fathers, an upright birthing position, and skin-to-skin to contact [12]. In contrast, a 2004 “birth guide”, compiled by surveying a convenience sample of women and hospital staff, revealed significant variations in the quality of information provided to pregnant women and in respectful treatment from staff [13].

Neither of these previous surveys explored the effects of informal cash payments, even though in Hungary more than 60% of pregnant women pay informally for birth care [14]. Informal and formal cash payments may affect quality of maternity care in terms of affordability and accessibility of services. As with fee-for-service official payment schemes, informal payments may generate unnecessary use of services with “doctors recommending procedures in order to increase their income rather than for therapeutic benefit [15, 16].” Even though quantitative data is lacking, qualitative studies from Serbia and the Ukraine found that women pay informally to have a “chosen” obstetrician attend their births, and that they perceive many benefits to this continuity relationship—mainly, receiving more respectful care [10, 17]. However, given the lack of representative data to measure and monitor the quality of maternity care, the extent to which women actually benefit from these payments is unknown.

Investigators in the United States and in Canada have used cross-sectional surveys to assess women’s experiences of quality maternity care, addressing issues of evidence-based care, doctor-patient communication, and the process of decision-making in birth. The American survey *Listening to Mothers 3* (LTM3) has been administered three times to a representative cohort of U.S. women [18]. *Changing Childbirth in British Columbia* (CCinBC) incorporated items from LTM3 but also used community-based participatory research methods to develop new items pertaining to women’s maternity care preferences, their decision-making, and perceptions of autonomy and respect [19, 20]. Given the existence of high-quality, English-language survey items, we decided to adapt and content validate these items for use in the Hungarian context with the primary aim of creating a comprehensive questionnaire to explore quality maternity care. Specifically, we examined quality care according to the rates of obstetric procedures, several measures of the experience of care, as well as the prevalence of informal cash payments.

## Methods

### Survey construction

To create the first version of the questionnaire, we combined the LTM3 and the CCinBC surveys. Duplicate items and those specific to foreign systems (e.g., American health insurance) were excluded. We added a validated scale to measure women’s role and ability to participate in decision-making, the Mothers Autonomy in Decision Making scale (MADM) developed by Vedam and colleagues [19]. We adapted informal payment questions from a cross-country survey on general inpatients [21].

The Hungarian maternity care system has similarities to Canada and the United States. A national health insurance scheme covers Hungarian maternity services (as in Canada), and a Hungarian woman has her choice of private or public prenatal providers (as in Canada and the U.S.). Like North America, providers are not required to be present for the births of their prenatal patients, in which case the “on-call” provider attends the birth. Unlike North America, in Hungary a pregnant woman who desires to have her “chosen” prenatal provider present at her birth will informally “contract” with her physician for the “extra” service of attending the birth [22].

Informal cash payments pose several challenges to quantitative exploration. First, informal cash payments are usually unregistered, and no government data source exists [23]. Second, Stepurko et al. found that respondents frequently refuse to answer questions about informal payments [24]. Finally, a woman typically pays after her delivery, making it challenging to explore associations with prenatal and birth outcomes that necessarily happen prior to the payment [17]. Thus, we needed to develop survey items that would be both culturally acceptable and—at least in concept—precede in time the outcomes of interest.

### Content validation

When designing instruments it is common to undertake a validation process to provide evidence that the instrument is relevant to the regional context. One approach is to have experts judge the relationship between the survey items and the theory on which the instrument is based [25, 26]. We invited 31 lay and professional maternity care content experts—including active users of the system—to validate the comprehensiveness and regional specificity of our questionnaire [27]. Experts were identified through purposive sampling of research, professional, and birth-advocacy networks to maximize non-overlapping expertise [28]. Those experts who accepted our invitation were instructed on how to review the survey items in light of the concept of “women-centered care”, focusing on issues of continuity of care, doctor-patient communication, care preferences, and the use of evidence-based techniques [3, 29]. We required all experts to be bilingual in English and Hungarian.

The final survey instrument, “The Mother-Centered Pregnancy Care Survey”, consisted of 111 items: 5 screening, 16 prenatal care, 35 birth care, 12 postpartum care, 22 care preferences, 11 informal payments, 8 MADM scale items, 2 open-ended questions that inquired about the best and worst aspects of the experience of care. Among these, a total of 75 questions collected information on elements of women-centered care and were woven across the above domains.

The final questionnaire then underwent 5-way independent translation, as has been first previously used in Hungarian research, consisting of 3 independent translators who worked in parallel, followed by 1 translator who reconciled and assembled these parallel versions, and concluded by 1 final back translation of the reconciled Hungarian version into English [30]. The final back translation was checked for accuracy by an author who is a native English speaker (NR). Four Hungarian maternity care users beta-tested the survey for language, length, clarity, and functionality.

### Survey administration

With the help of mostly international private donors without vested interests, a sum of $4300 US dollars was raised to retain the survey firm Ipsos. Ipsos maintains a panel of more than 70,000 members who are representative of Hungarian internet users for age, sex, and geographical location. We selected women between the ages of 18–45 with children under the age of 5 as the “target” population (total available *N* = 7762).

#### Sample

Ipsos administered the survey to the target population using a quota system to ensure a representative distribution regarding age, marital status, household size, education level, monthly income, settlement, and marital status. Balancing the resources available with the sample size needed to conduct a robust analysis, Ipsos stopped the invitations once a representative sample of 600 women was achieved. Ipsos also managed data collection for a convenience sample (*N* = 657) obtained via social media networks of birth and parenting organizations. Recruitment lasted for the month of October 2014. All respondents gave informed consent prior to initiating the survey.

#### Analysis

The research team reviewed all numeric and qualitative data supplied by the expert panel. We assessed inter-rater agreement by using the content validity index (CVI), summing the number of experts who rated an item as highly relevant and clear (level 3 or 4) and dividing by the total experts. We then averaged these scores to generate an item-level CVI (I-CVI). We considered items to be relevant and clear with an I-CVI score greater than 0.8 [26]. We reviewed all comments with equal attention, giving extra weight to repeat themes. Revisions were done carefully in dialogue between a native English speaker (NR) and a native Hungarian speaker (IS) in order to maintain clarity.

We compared demographic characteristics with two-tailed z tests (for dummy variables) or Pearson Chi^2^ test (for categorical variables). We then compared the amount of informal payments across groups by two-tailed t or z tests (ANOVA). We employed STATA version 14.1 for all statistical calculations. Responses to an open-ended question “What was the worst thing about the care you received during your recent birth?” underwent thematic analysis [31]. Two authors (ZS and ES) read through all the responses, categorized the content, and then coded the content by hand in order to determine the frequency of different themes.

The Regional Ethics Committee of Semmelweis University, Budapest (ref. number: 99/2014) approved this study. Because participation in the study was voluntary and preserved the anonymity of the participants with no invasive sampling techniques, the ethics committee did not require a formal consent process. Nonetheless, the survey opened with a discussion of risks, benefits, and potential harms, and then stated that by starting the survey a woman consented to participate. Our research was conducted in full accordance with the World Medical Association Declaration of Helsinki.

## Results

Eleven of the 31 invited multi-disciplinary experts completed the entire validation process. The final panel consisted of: research and a clinical psychologists [2]; obstetrician-gynecologists [2]; a lawyer expert in birth issues [1]; directors of non-governmental organizations [2]; a midwife [1]; a doula [1]; an epidemiologist [1]; a mother [1]. One of the psychologists runs a support group for new mothers, and the NGO directors lead initiatives on expanding pregnancy and birth options. The doula herself is a mother and has supported birthing mothers in Hungary. Thus, 6 of the 11 experts had personal experiences as, or close relationships with active users of Hungarian maternity care.

**Fig. 1:**
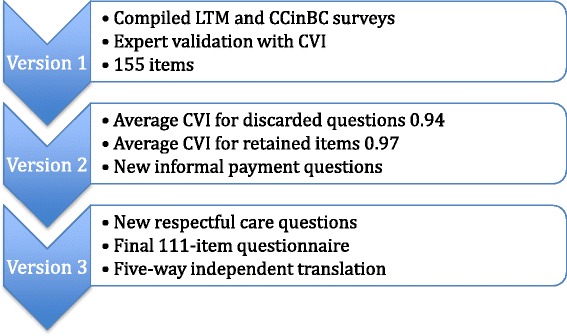
Survey development process

Figure 1 summarizes the survey development and validation process. Only 3 items scored below the commonly used I-CVI cut off at or below 0.8. The LTM3 question “Is your baby during this time period living?” received an I-CVI of 0.8. Experts felt this question used harsh language and might turn women away. Another question from LTM3, “Did you get your first prenatal visit as early in your pregnancy as you wanted?” scored 0.76; experts felt this question was not relevant to a socialized health system. As a group the informal payment questions received scores (I-CVI 0.93) above the cut off for inclusion. However, experts consistently commented on the lack of relevance to the intrapartum context of questions developed for general inpatients. Table 1 lists the nine new informal payment questions that we developed with expert input.

**Table 1:** Nine new informal cash payment questions

Considering all types of official and informal cash payments, how much IN TOTAL did you spend (out of pocket, in cash) related just to the delivery of your baby? This refers to the amount of cash paid after your prenatal visits concluded. a. Total amount b. I don’t remember c. Decline to answer	Beleértve az összes hivatalos díjat és nem hivatalos hálapénzt, mennyi pénzt fizetett Ön (és családja) összesen a szülésért a saját zsebéből, készpénzben? Ebbe az összegbe ne számítsa bele a szülést megelőző vizitek árát akkor sem, ha a befizetett összeg után nem kapott számlát! a. Teljes összeg: ezer FORINT b. Nem emlékszem. c. Nem kívánok válaszolni.
How much of this [X amount paid] for your birth was an informal cash payment? a. Total amount b. I don’t remember c. Decline to answer	Ebből az összegből a hálapénz összege: a. ezer FORINT b. Nem emlékszem. c. Nem kívánok válaszolni.
When you paid cash for your pregnancy and birth care--in the private or the public system--what did you expect to receive in return? Choose all that apply. I expected to…Yes/No (Select all that apply instead of Yes/No) a. Receive better quality care b. Obtain more attention from the staff c. Find a more skilled physician and/or midwife d. Wait less time to get an appointment e. Have better access to my doctor and/or midwife f. Have my chosen doctor or midwife attend my birth g. Have more control over my care h. Because I felt thankful for care I received. i. Get nothing in return. I felt is was required to pay. j. Other [text box]	Amikor hálapénzt fizetett a szülésért mit várt el a pénzéért cserébe? Több választ is bejelölhet. “Igen”-nel és “Nem”-mel felelhet. Azt vártam, hogy... a. jobb minőségű ellátást kapjak. b. több figyelmet kapjak a személyzettől. c. jobb orvost és/vagy szülésznőt kapjak. d. kevesebbet kelljen várnom arra, hogy időpontot kapjak. e. jobb hozzáférésem legyen az orvosomhoz és/vagy szülésznőmhöz. f. a választott orvosom vagy szülésznőm legyen jelen a szülésemnél. g. legyen beleszólásom az ellátásomba. h. Azért fizettem, mert hálás voltam az ellátásért, amit kaptam. i. Nem vártam semmit viszonzásképpen. Muszáj volt fizetnem. j. Egyéb: a fentiektől eltérő dolgot vártam:
When did you make the informal cash payment for your delivery? a. Before I gave birth b. After I gave birth while I was in the hospital c. After I gave birth and went for a visit to the clinic d. I don’t remember	Mikor fizetett Ön hálapénzt a szüléséért? a. A szülés előtt. b. A szülés után, amíg még a kórházban voltam. c. A szülés után, amikor vizsgálatra/ellenőrzésre mentem. d. Nem emlékszem.
You spent [x amount] for all of your prenatal visits and birth care, formal and informal. Was it necessary to borrow cash from family or friends, the bank or from a credit card, or sell personal assets to cover this cost? a. Yes b. No c. I don’t remember	Ön összesen (beleértve a hivatalos összegeket és a hálapénzt is) forintot költött a várandósgondozásra és a szülésre. Kellett ehhez kölcsönkérnie pénzt családtagoktól vagy barátoktól, esetleg banki kölcsönt felvennie vagy hitelkártyán túlköltenie, vagy eladni valamilyen személyes tárgyat/tulajdont, hogy ki tudja fizetni ezt az összeget? a. Igen. b. Nem. c. Nem emlékszem.
You paid an informal payment during your prenatal care or for your birth. Did your provider ask you to pay a specific amount, or did they leave it up to you to decide how much to pay? a. Yes, they asked for a specific amount. b. No, they let me decide how much to pay. c. I don’t remember	Amennyiben fizetett hálapénzt a várandósgondozás alatt és a szülésért, kérte Öntől az szülészeti ellátója, hogy fizessen egy bizonyos összeget vagy Önre bízta, hogy mennyit fizet? a. Igen, kértek egy bizonyos összeget. b. Nem, rám bízták, hogy mennyit fizetek. c. Nem emlékszem
You paid [x] amount in total for informal cash payments during your prenatal care and birth. To whom did you make this informal cash payments? Choose all that apply. a. A doctor in the clinic b. A nurse in the clinic c. A doctor in the hospital d. A nurse in the hospital e. A midwife in the hospital f. A midwife at home g. Other h. I don’t remember	Ön ezer forint hálapénzt fizetett a várandósgondozásért és a szülésért. Kinek adta ezt a hálapénzt? Jelölje be azokat a személyeket, akinek adott pénzt! Több személyt is megjelölhet. a. Egy orvosnak a rendelőben. b. Egy nővérnek a rendelőben. c. Egy orvosnak a kórházban. d. Egy nővérnek a kórházban. e. Egy szülésznőnek a kórházban. f. Egy otthonszülést kísérő bábának. g. Másnak. h. Nem emlékszem.
You said that you paid [x amount] in informal cash payments for your pregnancy and birth care. How did you feel about this informal cash payment? Very negative/ Somewhat negative/ Indifferent/ Somewhat positive/ Very positive	Amennyiben fizetett hálapénzt a várandósgondozás alatt és a szülésért, hogyan érintette Önt, hogy fizetnie kellett? Nagyon rosszul érintett / Kicsit rosszul érintett / Közömbösen / Meglehetősen pozitívan érintett / Nagyon pozitívan érintett
During your recent birth while in the hospital or at home, how often were you treated poorly because of…? Check all that apply. a. Your race, ethnicity, cultural background or language spoken b. Your financial situation c. Your sexual orientation or gender identity d. You refused care that your provider recommended e. Because you developed a birth plan f. Because you did not pay an informal cash payment Never/ Sometimes/ Usually/ Always	A legutóbbi vajúdásánál és szülésénél - akár kórházban zajlott, akár otthon - milyen gyakran bántak Önnel igazságtalanul az alábbi okokból? Jelölje meg az összeset, amely igaz. Több választ is bejelölhet. a. Az Ön bőrszíne, nemzetiségi hovatartozása, kultúrális háttere, anyanyelve miatt? b. Az Ön anyagi helyzete miatt? c. Az Ön szexuális orientációja vagy nemi identitása miattt? d. Azért, mert Ön visszautasította a szülészeti ellátója javaslatait? e. Azért, mert Ön szülési tervvel érkezett? f. Azért, mert Ön nem adott hálapénzt? Soha/néha/általában/mindig

After the informal payment questions the next most challenging group of items referred to the overlap between payments and the model of care (doctor, midwife). Experts decided that the process of paying a provider informally hinged on the model that the majority of women select prior to delivery. In accordance with previous research and their own system knowledge, experts then decided to use the word “chosen” (*választott*) to refer to the continuity prenatal relationship that women pay for informally. The word “chosen” was then applied to the private and public models of care to yield three models of chosen doctor care and one model of chosen (hospital) midwifery care. Two models of “not chosen” care represented the default model provided by the state insurance system. Independent home birth midwifery was its own category. These linguistic results are shown in Table 2.

**Table 2:** Model of care categories with linguistic results

Which of these providers was the most important source of your prenatal care?	Hungarian linguistic adaptation of model of care categories	Convenience N = 657 (%)	Representative N = 600 (%)
Chosen doctor in a private hospital system	választott orvos magánkórházban	10 (1.5)	2 (0.3)
Chosen doctor in a private practice	választott orvos magánrendelésen	287 (43.8)	167 (28.0)
Chosen doctor in a state institute	választott orvos állami intézményben	119 (18.1)	184 (30.8)
Chosen hospital midwife	választott (kórházi) szülésznő	78 (11.9)	28 (4.7)
Independent (home birth) midwife	független bába	82 (12.5)	3 (0.5)
I did not choose a doctor, just went to my local clinic	nem választottam orvost, a helyi rendelőintézetbe/szakrendelőbe jártam	68 (10.4)	155 (26.0)
District public health nurse	védőnő	12 (1.8)	58 (9.7)
I did not go to prenatal care	nem jártam várandósgondozásra	1	3 (0.5)

Ipsos field tested the survey and confirmed that the duration of participant engagement required approximately 30 min. Altogether, Ipsos sent 892 e-mail invitations to their panel with a response rate of 67%. Reasons for drop out were: 14 (1.6%) quota full, 115 (12.9%) screened out, 163 (18.3%) terminated the survey. In addition, 657 completed surveys were obtained through convenience internet sampling. Table 3 shows demographic indicators for the sample with the corresponding most recent census data listed below the table. Overall, the representative sample compared well to recent census data. Pearson Chi^2^ statistics show that the convenience sample was statistically significantly more highly educated women (Chi^2^ = 341.8, *p* < 0.0001), lived in the capital (Chi^2^ = 128.2, *p* < 0.0001), and had higher average net incomes (*t* = −16.02, *p* < 0.0001).

**Table 3:** Social demographic indicators

		Convenience *N* = 657	Representative *N* = 600
Age	Age in years (SD) Min, Max	33.7 (4.18) 20, 47	33.3 (4.96) 21, 45
Education^a^(%)	Less than <7 grade	0	17 (2.8)
	Grade 8	0	16 (2.7)
	Trade School	9 (1.3)	92 (15.3)
	High School	91 (13.9)	244 (40.7)
	College	256 (39.0)	167 (27.8)
	University diploma	301 (45.8)	64 (10.7)
Settlement^b^(%)	Capital	304 (46.3)	100 (16.7)
	County Seat	95 (14.5)	124 (20.7)
	City	151 (23.0)	200 (33.3)
	Village	107 (16.3)	176 (29.3)
Net Income^c^ (thousands HUF)	Mean (SD)	374 (218)	209.23 (118)
	Max	2250	875
	Missing		39 (6.5)

^a^Census data education, women age 20–49: Less than high school 19.6%; completed high school 39.5%; college degree and above 26.1%

^b^Census data settlement, entire population: Capital 17.4%; County seat 20.4%; city 31.7%; village 30.5%

^c^Census data income, net household 2014: average 158 thousands of forints

Table 4 reveals the informal payment practices in the representative sample according to the model of care categories. Excluding the categories with fewer than five respondents, we see that the response percentages to the informal payment question ranged between 75 and 86%. Pearson Chi^2^ statistics showed that the share of women who paid informally was significantly different across the groups (Chi^2^ = 183.6; *p* < 0.0001). ANOVA test shows that the amount of informal payment is also significantly different across groups (F = 6.73, *p* < 0.0001).

**Table 4:** Informal payments by provider type, representative sample

	Answered informal payment question, *N* (%)	Reported paying informally, *N* (%)	Av. amount of informal payment EUR (SD)
Chosen doctor in a private hospital system	2 (100)	2 (100)	333 (236)
Chosen doctor in a private practice	125 (75)	102 (82)	210 (128)
Chosen doctor in a state institute	138 (75)	108 (78)	169 (103)
Chosen hospital midwife	24 (86)	22 (92)	203 (99)
Independent (home birth) midwife	3 (100)	0 (0)	–
I did not choose a doctor, just went to my local clinic	125 (81)	21 (17)	81 (45)
District public health nurse	50 (86)	10 (20)	118 (65)
I did not go to prenatal care	2 (67)	0 (0)	–
Total	469 (78)	265 (57)	180 (116)

Regarding informed consent practices, Table 5 shows the responses from the representative sample as to whether a woman’s permission was obtained prior to undergoing a cesarean (*N* = 244) or an episiotomy (*N* = 257). Pearson Chi^2^ statistics showed that the permission practices were significantly different across provider types for cesarean section (Chi^2^ = 39.2, *p* = 0.003) but were not significantly different for episiotomy (Chi^2^ = 18.6, *p* = 0.414). MADM scores were significantly different across permission categories (ANOVA results for caesarean: F = 14.50, *p* < 0.0001, for episiotomy: F = 10.34 *p* < 0.0001).

**Table 5:** Permission for cesarean (*N* = 244) or episiotomy (*N* = 257), representative sample

	Yes, they asked and I gave my permission.		No, they did not ask my permission.		I refused the procedure, but they still did it.		I don’t remember	
	Cesarean	Episiotomy	Cesarean	Episiotomy	Cesarean	Episiotomy	Cesarean	Episiotomy
Chosen doctor in a private practice (%)	72 (91.1)	25 (35.2)	5 (6.3)	42 (59.2)	0	1 (1.4)	2 (2.5)	3 (4.2)
Chosen doctor in a state institute (%)	79 (90.8)	27 (35.5)	6 (6.)	45 (59.2)	0	0	2 (2.3)	4 (5.2)
Chosen hospital midwife in a private or state clinic (%)	4 (57.1)	7 (41.2)	2 (28.6)	9 (52.9)	0	0	1 (14.3)	1 (5.9)
I did not choose a doctor, just went to my local clinic (%)	37 (72.6)	16 (24.2)	9 (17.7)	46 (69.7)	1 (2.00)	1 (1.5)	4 (7.8)	3 (4.6)
District public health nurse (%)	14 (70.0)	8 (29.6)	6 (30.0)	18 (66.7)	0	0	0	1 (3.7)
Total (%)	206 (84.5)	83 (32.6)	28 (11.4)	160 (62.0)	1 (0.4)	2 (0.8)	9 (3.7)	12 (4.7)
MADM score Mean (SD)	26.9 (7.5)	28.1 (6.5)	19.0 (5.7)	22.3 (8.3)	–	23.5 (12.0)	18.4 (7.1)	23.7 (7.9)

Table 6 shows the coded results from an open-ended question from LTM3. Thematic analysis of the open-ended responses (*N* = 1015) from the entire sample identified 13 priority areas of the maternity care experience, 9 of which were addressed by the questionnaire.

**Table 6:** Thematic analysis of responses to open-ended question: What was the worst thing about the care you received during your recent birth? (*N* = 1015)

	Explored by any items in the final survey
1. No consent for interventions / interventions done against my wishes	Yes
2. Painful interventions (vaginal examinations, cervix stretching, episiotomy)	Yes
3. Doctor/midwife style	Yes
4. Hurrying the labor	Yes
5. I could not choose a comfortable position	Yes
6. They did not help with breastfeeding	Yes
7. Lacking information	Yes
8. Did not allow support people to be present	Yes
9. Problems with prenatal care	Yes
10. Hospital condition (room, bed, food, bathroom)	No
11. Newborn hospital unit	No
12. Children could not be with me	No
13. Told home birth was too dangerous	No

## Discussion

We used a standardized and rigorous methodology to develop and validate a survey instrument that comprehensively examined maternity care experiences in Hungary, thus filling an important gap where no government-sponsored data exists. The process included collating validated items from the international literature, adapting them to the Hungarian context by expert panel, designing region-specific new items, and validating the content. To the best of our knowledge, no other group has undertaken this task. We found that existing English-language survey items concerning the experience of maternity care were clear and relevant to the Hungarian context. This is likely due to the fact that many of the issues related to excessive obstetric procedures, poor communication, and the lack of maternal autonomy that we found in Hungary are also common in the United States and Canada [8, 18, 19].

Our expert process proved effective at identifying survey domains that required additional adaptation. For example, our maternity care experts identified that informal payment questions developed for general inpatients required adaptation. Our expert panel integrated linguistic, system, and user expertise to develop new survey items specific to the CEE region. We believe this was a result of the collaboration across our diverse panel. Some argue that content experts should have significant research or clinical experience. However, inclusion of “lay” experts has been found to be appropriate in many situations [27] and is consistent with the principles of patient-centered research [32].

To test reliability, we administered the survey to two samples of service users: a randomly selected representative sample and a parallel convenience sample. The instrument performed well in both groups: it was user friendly, feasible to distribute in an online format, and captured information on several domains relevant to maternal experience of care during pregnancy and childbirth.

In our 30-min survey 67% of the items addressed issues of person-centered care. We found that the extent of informed consent and autonomy (MADM scores) varied significantly across the model of care categories. We also found lower MADM scores in the women who had cesareans and episiotomies performed without their consent. Lack of consent for procedures was a common theme in the responses to the open-ended question. These findings are discussed in detail in a separate paper [33] and are supported by qualitative studies that show that women pay informally to receive care that they perceive to be more respectful [10, 17]. Analysis of our model of care categories showed extensive overlap between informal payments and the use of the word “chosen”. Women who went to their local clinic *without* choosing a doctor paid informally 17% of the time—the lowest frequency of all the models of care. We believe the statistically significant different distribution of informal payments across the care categories validates these categories for future research in Hungary and the CEE region. Because informal payments may distort health care services in ways that require policy intervention [21], reliable survey items are necessary to evaluate their effects [23].

### Limitations

Because we chose an expert validation process without extensive community involvement, we may not have addressed additional elements of mother-centered care in this population. For example, responses to the open-ended questions indicate additional items could have addressed the physical state of the maternity and newborn wards, newborn care in general, and home birth. Additionally, internet users may not be representative of the Hungarian general population; a more representative sample would require telephone or face-to-face interviewing. Finally, given the challenges of surveying the broad preferences and outcomes of the entire maternity system, ideal distribution of our survey would capture more pathways, especially for ethnic/minority and poor women.

## Conclusion

We developed a reliable and relevant survey instrument to evaluate evidence-based care and maternal experiences in Hungary. This survey instrument can be easily adapted for use in other Central and Eastern European countries, where informal payments, the variable application of evidence, and concerns with respectful provider-patient relationships are similar. We plan to utilize the data resulting from this survey to inform interprofessional education and elucidate determinants of high quality maternity care in Hungary. A survey similar to ours could be used to regularly monitor trends in Hungarian maternity care as well as for cross-country comparisons in the CEE region, where representative data on quality maternity care is lacking.

## Abbreviations

CCinBC: Changing Childbirth in British Columbia
CEE Region: Central and Eastern European Region
CVI: Content Validity Index
LTM3: Listening to Mothers 3
MADM: Mothers Autonomy in birth Decision Making scale
